# Production of *β*-Glucosidase from a Newly Isolated *Aspergillus* Species Using Response Surface Methodology

**DOI:** 10.1155/2011/949252

**Published:** 2011-04-18

**Authors:** Pilanee Vaithanomsat, Molnapat Songpim, Taweesiri Malapant, Akihiko Kosugi, Warunee Thanapase, Yutaka Mori

**Affiliations:** ^1^Enzyme Technology and Waste Management Research Unit, Kasetsart Agricultural and Agro-Industrial Product Improvement Institute (KAPI), Kasetsart University, 50, Chatuchak, Bangkok 10900, Thailand; ^2^Post-Harvest Science and Technology Division, Japan International Research Center for Agricultural Sciences (JIRCAS), 1-1, Ohwashi, Tsukuba, Ibaraki 305-8686, Japan

## Abstract

A newly isolated fungus *Aspergillus niger* SOI017 was shown to be a good producer of *β*-glucosidase from all isolated fungal strains. Fermentation condition (pH, cellobiose concentration, yeast extract concentration, and ammonium sulfate concentration) was optimized for producing the enzyme in shake flask cultures. Response surface methodology was used to investigate the effects of 4 fermentation parameters (yeast extract concentration, cellobiose concentration, ammonium sulfate concentration, and pH) on *β*-glucosidase enzyme production. Production of *β*-glucosidase was most sensitive to the culture medium, especially the nitrogen source yeast extract. The optimized medium for producing maximum *β*-glucosidase specific activity consisted of 0.275% yeast extract, 1.125% cellobiose, and 2.6% ammonium sulfate at a pH value of 3.

## 1. Introduction

Lignocellulose is the major component of biomass, representing the most abundant renewable organic source in soil. It consists of three polymers types, cellulose, hemicelluloses, and lignin that are strongly intermeshed and chemically bonded by noncovalent forces and by covalent cross-linkages [1]. The enzymatic hydrolysis of cellulosic material into glucose involves the synergistic action of at least three different enzymes: endoglucanase or endo-*β*-1,4-glucanase, exoglucanase or exocellobiohydrolase, and *β*-1,4-glucosidase or cellobiase [2]. Endo-*β*-1,4-glucanase catalyzes the hydrolysis of cellulose by randomly splitting the sugar residues within the molecule, whereas exo-*β*-1,4-glucanase removes monomers and dimmers, from the end of the glucan chain. The *β*-1,4-glucosidase hydrolyzes glucose dimers and in some cases, cellulose oligosaccharides to glucose. Since cellobiose inhibits the action of endo- and exoglucanases, *β*-glucosidase contributes to the efficiency of this process. Very strong activity of *β*-glucosidase is thus needed for the pretreatment step of lignocellulose before a further ethanol conversion. In addition to the role in cellulose degradation, *β*-glucosidase has also been attributed to several other applications. This includes the applications in pharmaceutical, cosmetic, and detergent industries [3]. Most of the commercially available *β*-glucosidase are served as parts of cellulase enzymes. There are only two of those served as the actual *β*-glucosidase; one is the Novozym188 from *Aspergillus niger* (Novo Industri A/S, Denmark) and another one is *β*-glucosidase also from *A. niger* (Fluka, Switzerland). More screenings have been attempted for the novel sources of much higher *β*-glucosidase activity. Vaithanomsat et al. [4] isolates *A. niger* SOI017 with equivalent capability of *β*-glucosidase production to those two strains. Even though it is *A. niger* as the two commercial ones, it is worthed to more deeply studied whether there are differences among them. In order to achieve the purpose, high production of *β*-glucosidase is essential. 

Production of many enzymes by fermentation is highly susceptible to the composition of the culture medium and factors such as feedback inhibition by the carbon source. Therefore, identifying the medium composition to maximize the enzyme titer in a minimally controlled batch fermentation is important. Traditionally, fermentation processes have been optimized by changing one independent variable or factor at a time while keeping the others at some fixed values. This single dimensional search is slow and laborious, especially if a large number of independent variables are involved. Furthermore, the traditional optimization does not reflect the interaction effects among the variables employed and does not depict the net effect of the various factors on the enzyme activity. Consequently, statistical methods are increasingly preferred for fermentation optimization because they reduce the total number of experiments needed and provide a better understanding of the interactions among factors on the outcome of the fermentation [5]. Statistical techniques such as the response surface methodology (RSM) have gained broad acceptance in fermentation optimization. RSM allows calculation of the optimum levels of various process parameters based on a few sets of experiments.

Here, the focus is on optimizing the production of the enzyme *β*-glucosidase by a newly isolated *A. niger* SOI017 using a low-cost minimally controlled batch fermentation process. The fermentation condition and the medium composition are optimized using the response surface methodology.

## 2. Materials and Methods

### 2.1. Selection of Strains

All collected samples were screened for their ability to grow and produce *β*-glucosidase in cellobiose-containing liquid media (0.1% yeast nitrogen base w/o ammonium sulfate, 0.5% yeast extract, 0.6% ammonium sulfate, and 2.0% cellobiose) as already described in [4]. The genus was identified by morphological observation as well as by molecular method [6]. The selected unknown species (SOI017) was grown on PDA at 30°C for 2 days. Approximately 50–100 mg of fresh fungal mycelium was scraped from cultures into a new microcentrifuge tube containing 20–60 *μ*L of 0.5 N NaOH including +70 meshes sand. 2–5 *μ*L of liquid suspension was transferred into a new microcentrifuge tube and 198 *μ*L Tris HCl, and pH 8 was added [7]. Finally, 3-4 *μ*L of this solution was used for the PCR reactions. A standard amplification protocol was followed with universal primers ITS5 (5′ GGA AGT AAA AGT CGT AAC AAG G 3′) and ITS4 (5′ TCC TCC GCT TAT TGA TAT GC 3′) described by White et al. [8] for the Internal Transcribed Spacers (ITS) regions while the Large Subunit (LSU) ribosomal RNA was amplified with LROR (5′ ACC CGC TGA ACT TAA GC 3′) and LR7 (5′ TAC TAC CAC CAA GAT CT 3′) described by [9, 10]. 

PCR was performed in a single 50 *μ*L PCR reaction mixture containing 39 *μ*L sterile deionized water, 5 *μ*L of 10X PCR buffer, 2.5 *μ*L of 50 mM MgCl_2_ (final concentration 2.5 mM), 1 *μ*L of 10 mM dNTP mix (final concentration 0.2 mM), 1 *μ*L of 10 *μ*M of each primer (final concentration 0.2 *μ*M), 0.5 *μ*L of Taq polymerase 1 unit/*μ*L (Promega Corp., Madison Wisconsin, catalogue no. M1665), 50 mM MgCl_2_, and 1 *μ*L genomic DNA (approximately 10 ng). PCR thermal cycling profile for amplifications of LSU and the 5.8S rDNA including flanking ITS regions consisted of denaturation step at 95°C for 2 min followed by 35 cycles 95°C for 1 min 55°C for 1 min, 72°C for 2 min, and the final step of 72°C for 7 min [11]. The PCR products were analyzed by gel electrophoresis (1.5% agar) and observed under UV-light after staining in ethidium bromide (0.5 ug/mL) for 15 minutes. Amplification product was purified using *Nucleospin Plant DNA purification kit* (Macherey-NAGel, catalogue no. 740 590.50). Purified PCR products were submitted for sequence determination by BIOTEC Service Unit (BSU) carried out in an automated sequencer. The consensus sequences for each species were multiple aligned by Clustal W 1.6 [12] along with other sequences obtained from the GenBank database. The dataset was refined visually in Se-Al v1.Oa1 and Bioedit 5.0.6 [13]. BLAST (basic local alignment search tool) was used to search for sequences similar to their ITS rDNA of *Aspergillus* strains from the National Center for Biotechnology Information (NCBI). Nucleotide BLAST search with the option standard nucleotide-nucleotide BLAST of BLASTN 2.26 was used to compare and find similar sequences [14]. In case of LSU, BLAST search was searched for sequences GenBank having high scores of percentage identity of *Aspergillus* sequences to test family and genus level [14]. For the ITS region, pairwise alignment in BioEdit program [13] was used to calculate exact position on nuclear partial 18S, ITS 1, 2 regions, 5.8 rDNA, nuclear partial 28S rDNA, and percentage of identity between sequences. The data also were analyzed by the Clustal W [12] and PAUP phylogenetic analysis programs [15]. Including currently corresponding sequences of representative *Aspergillus*, strains were included in the datasets for phylogenetic analyses. The phylogenetic trees were generated using neighbor-joining (NJ) criteria for analyses of nucleotides. *Rhizopus microsporous* (EF 134626) was chosen as the out-group taxa for all analyses. A neighbor-joining (NJ) tree was constructed. The stability of clade was evaluated by bootstrap tests with 1000 replications in distance analysis (neighbor-joining method). Bootstrap analysis using the heuristic search option of PAUP will be performed to calculate the robustness of each branch. The analysis was set as the following parameter: 1000 bootstrap replicates, with gaps treated as missing data, tree-bisection-reconnection branch-swapping algorithm and random sequence addition.

### 2.2. Microorganisms and Cultivation

The fungal stock cultures were maintained through a periodic transfer on Potato Dextrose Agar (PDA) at 4°C until use. To prepare the inoculum, the fungus was transferred onto a fresh PDA slant and incubated at 30°C for 7 days. The spores harvesting from the slants was performed using 5 mL sterile distilled water. This was ready to be used for further experiments.

### 2.3. Production of *β*-Glucosidase

The base medium (30 mL; g/L: 10.0 cellobiose; 5.0 yeast extract; 1.0 yeast nitrogen base (w/o amino acid and ammonium sulphate); 6.0 (NH_4_)_2_SO_4_; pH 5.5) was inoculated with 10% spore suspension (1 × 10^6^ spores) and cultured at room temperature on a rotary shaker (120 rpm). After 10 days of incubation, each flask was assayed for *β*-glucosidase activity (pNPG), total protein (Lowry's method), and substrates consumption (DNS).

### 2.4. Experimental Design

The RSM was used to investigate the effects of independent variables: agitation rate, temperature, and initial pH of medium on the responses of *β*-glucosidase activity. All treatment combinations (Table 2) were performed in 125 mL Erlenmeyer flasks. The experiments were performed according to the central composite design (CCD; Table 2). Using CCD for 4 factors (*k* = 4), 30 treatment combinations were generated. To set up a statistical model, five levels for each variable were chosen. The upper and lower limits of each variable were chosen to encompass the range in literature and to reflect what was done in practice after a preliminary investigation of the limits. The codes of ±*α* (±2.0) were designed at a distance of 2.0 (2^*n*/4^ = 2.0 for *n* = 4) from the design center. The remaining levels were identified using CCD.  Table 1 contained the actual factor levels corresponding to the coded factor levels as follows: *X*_1_ = (%cellobiose-1.75)/0.625, *X*_2_ = (%yeast extract-0.5)/0.225, *X*_3_ = (% (NH_4_)_2_SO_4_-1.6)/0.5 and *X*_4_ = (pH-6.0)/1.5. Table 2 showed the treatment combinations and mean response. From the experimental data according to this design, a second-order polynomial regression model was

(1) $$\begin{array}{cc} Y & =b_{0}+\;b_{1}X_{1}+\;b_{2}X_{2}+\;b_{3}X_{3}+\;b_{11}{X^{2}}_{1}+\;b_{22}{X^{2}}_{2}\\ & \;+b_{33}{X^{2}}_{3}+b_{12}X_{1}X_{2}+b_{13}X_{1}X_{3}+b_{23}X_{2}X_{3}+£, \end{array}$$

where *Y* = *β*-glucosidase activity (U/mL), *b*_*i*_ = the linear coefficients, *b*_*ii*_ = the quadratic coefficient, *b*_*ij*_ = the cross product coefficients, and *£* = the model constant.

### 2.5. Enzyme Activity

*β*-glucosidase was assayed using 50 *μ*L of appropriately diluted culture filtrate: The 250 *μ*L of sodium acetate buffer (100 mM, pH 5.0), 250 *μ*L p-nitrophenyl-*β*-D-glucopyranoside (pNPG) (4 mM, Sigma). After incubation at 60°C for 10 min, the reaction was interrupted by adding 2 mL of sodium carbonate 2 M, and the color was measured at 410 nm. One unit of *β*-glucosidase corresponds to the amount of enzyme that releases 1 *μ*M nitrophenol per min in the reaction mixture [2].

## 3. Results and Discussion

### 3.1. Microorganism Identification

**Figure 1:**
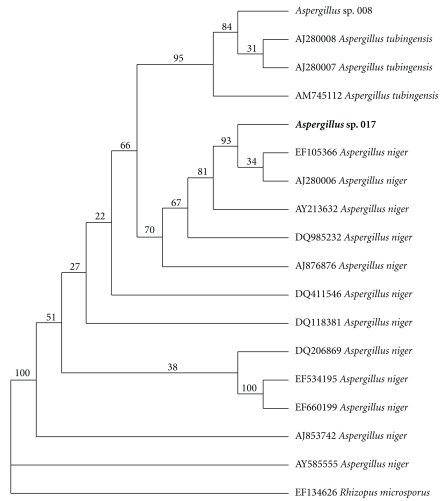
NJ trees calculated from ITS1-2, 5.8S rDNA sequence. *Rhizopus microsporus* was used as outgroup taxa. The internal topology of *Aspergillus* sp. 017 was grouped to *Aspergillus niger* (EF105366 and AJ280006) with the 93% supportive bootstrap branch.

The 5.8S gene of the rRNA and the two intergenic spacers ITS1 and ITS2 of SOI017 were sequenced and analyzed. The BLAST search result based on ITS region revealed that the genus *Aspergillus* SOI017 was identified to *A. niger *(Figure 1, Table 3), which is in the family Trichocomaceae, order Eurotiales, subclass Eurotiomycetidae, class Eurotiomycetes, and phylum Ascomycetes (http://www.ncbi.nlm.nih.gov/BLAST/). Additionally, the LSU result displayed that the *Aspergillus *SOI017 matched with *A. niger* CBS 513.88, complete genome (ACCESSION: NW001594105 and NW001594104).

### 3.2. Optimization of *β*-Glucosidase Production by RSM

The objective of the experimental design was to optimize the reaction conditions for maximizing the enzyme yield. Since optimization by one factor at a time method or the full factorial method cannot examine all the possible combinations of independent variables or is too laborious to perform, respectively, the appropriate statistical experiment design tools for optimization are important. In this study, the RSM was used for finding out the optimum condition for *β*-glucosidase production. After the experiments according to all treatment combinations, the data were analyzed using SPSS for Windows to yield regression (1), regression coefficients, and analysis of variance. From the analysis of variance (ANOVA), the model of specific activity *β*-glucosidase was highly significant (*P* < .05; Table 4) and the *R* ^2^ value for the model, being the measure of the goodness of fit of the model, was 0.709, which indicated that 70.90% of the total variations in the observed response values could be explained by the model, or by experimental parameters and their interactions. The rest (29.10%) of the total variation was not explained by the model. Coefficient estimates in the regression model for *β*-glucosidase was presented in Table 5. 

After the treatment combinations, all linear terms of the independent variables, quadratic term of %yeast extract, and interaction terms of %yeast extract with %yeast extract and %cellobiose with %cellobiose were included in the model for *β*-glucosidase production since these were significant (*P* < .05). The *P* value was used as a tool to check the significance of each of the coefficients. The smaller the magnitude of *P* value, the more significant was the correlation with the corresponding coefficient. Among the 4 factors tested, %yeast extract had the highest impact on specific enzyme activity as given by the highest linear coefficient (17046.43, Table 5). Thus, in the enzyme activity of* A. niger* SOI017, %yeast extract was the most important parameter. The model equations for *β*-glucosidase activity with the coefficients in coded units of factors were given in Table 6.

**Figure 2:**
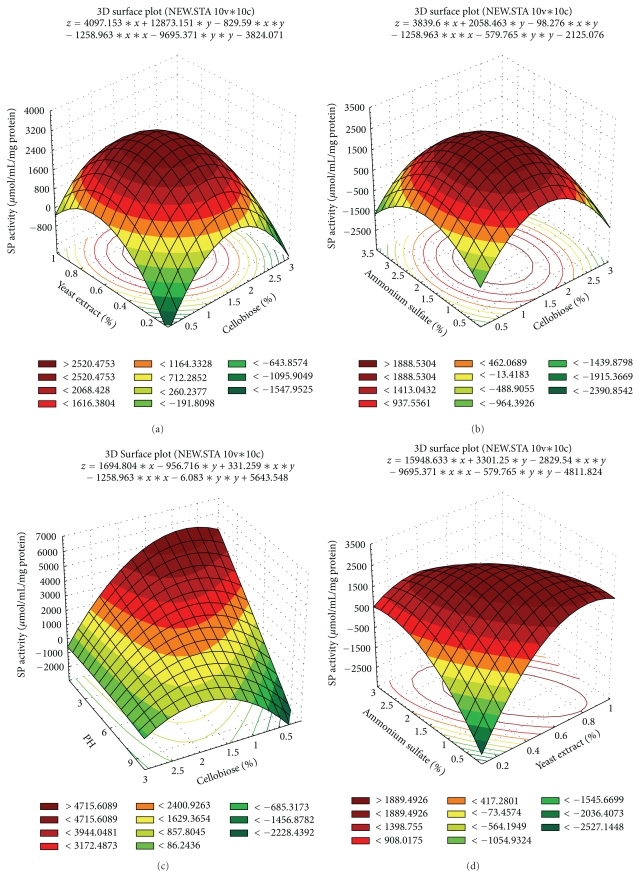

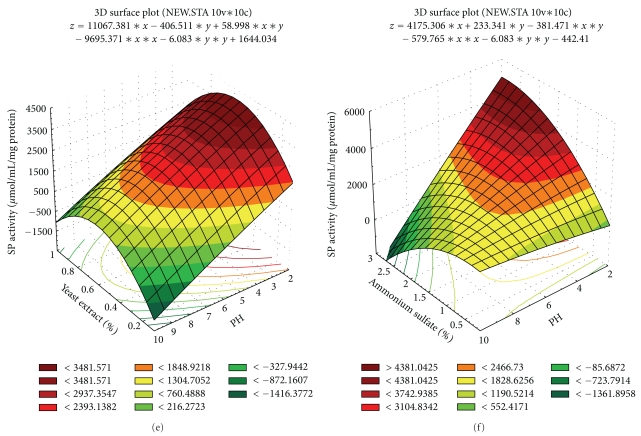
(a) Response surface for the effects of cellobiose (%) and yeast extract (%) at 1.6% (NH_4_)_2_SO_4_ and pH 6 on the specific activity after 240 h of incubation. (b) Response surface for the effects of cellobiose (%) and (NH_4_)_2_SO_4_ (%) at 0.5% yeast extract and pH 6 on the specific activity after 240 h of incubation. (c) Response surface for the effects of cellobiose (%) and pH at 1.6% (NH_4_)_2_SO_4_ and 0.5% yeast extract on the specific activity after 240 h of incubation. (d) Response surface for the effects of yeast extract (%) and (NH_4_)_2_SO_4_ (%) at 1.75% cellobiose and pH 6 on the specific activity after 240 h of incubation. (e) Response surface for the effects of yeast extract (%) and pH at 1.75% cellobiose and 1.6% (NH_4_)_2_SO_4_ on the specific activity after 240 h of incubation. (f) Response surface for the effects of (NH_4_)_2_SO_4_ (%) and pH at 1.75% cellobiose and 0.5% yeast extract on the specific activity after 240 h of incubation.

The effect of interaction of various parameters on the *β*-glucosidase production was studied by plotting three-dimensional response curves against any two independent variables while keeping the other independent variable at their “0” levels as shown in Figure 2. In predicting the response, all three-dimensional response surface graphs were generated using STATISTICA for Windows (Release 5.0, Stasoft, USA).  Figure 2(a) depicts three-dimensional curve of the calculated response surface from the interaction between %yeast extract and %cellobiose while keeping pH and % (NH_4_)_2_SO_4_ at “0” level. The response surface plot indicated an optimum *β*-glucosidase-specific activity around 0.6–0.8% yeast extract and 1.5–2.0% cellobiose. Increasing yeast extract concentration beyond 0.8% negatively affected *β*-glucosidase production. A similar inhibitory effect of the higher levels of yeast extract on *β*-glucosidase production by *Paecilomyces variotii *MG3 and *Debaryomyces pseudopolymorphus* UCLM-NS7A was also reported by Job et al. [3] and Barbosa et al. [16], respectively. This observation may indicate the sensitivity of *β*-glucosidase production by *A. niger *SOI017 to the nitrogen level in the medium. The concentration of carbon source also had the effect on *β*-glucosidase production by *A. niger *SOI017 as demonstrated in Figures 2(a), 2(b), and 2(c). Moussa and Tharwat [17] suggested the possibility of *β*-glucosidase production induced by cellobiose. Since the polymeric substrates are unable to cross the cell plasma membranes, the cells receive the signal for an accelerated synthesis of secreted glycanases by means of low-molecular weight fragments, usually disaccharides, derived from the polysaccharides. The fragments are formed by the action of small amounts of the enzymes produced constitutively. Thus, cellobiose could be an inducer for cellulose-degrading enzymes including *β*-glucosidase. Furthermore, the decrease in *β*-glucosidase production was observed when cellobiose concentration was higher than 2.0%. It may be suggested that the *β*-glucosidase production by *A. niger *SOI017 was repressed by easily metabolized sugars such as glucose [17]. In this case, glucose (the end product of cellobiose) interacted with a cellular protein and formed a complex which interacted with a particular gene at the transcription level and repressed cellulose synthesis [18]. When the pH and %yeast extract were fixed at 6 and 0.5% (“0” level), a maximum *β*-glucosidase-specific activity was obtained at 1.5–2.0% cellobiose and 2.0–2.5% (NH_4_)_2_SO_4_ (Figure 2(b)). Since Figure 2(c) shows the interaction of pH and %cellobiose at 1.6% (NH_4_)_2_SO_4_ and 0.5% yeast extract (“0” level), it indicated that the maximum *β*-glucosidase-specific activity was achieved around pH 3–5 and 1.5–2.0% cellobiose. When pH and %cellobiose were fixed at 6 and 1.75%, respectively, a maximum *β*-glucosidase-specific activity was observed at around 0.8–1.0% yeast extract and 2.5–3.0% (NH_4_)_2_SO_4_ (Figure 1(d)). Taken all together, high *β*-glucosidase-specific activity was achievable. 

In order to validate the obtained model, randomly selected six experiments from the various solutions provided by the software were performed, and the *β*-glucosidase activities were determined. Correlation analysis was performed on the actual responses obtained and the predicted values for each solution (Table 7). The optimum condition obtained from the RSM model was 0.275% yeast extract, 1.125% cellobiose, and 2.6% (NH_4_)_2_SO_4_ at a pH value of 3. Through the optimization of process parameters, we could obtain a twofold increase in the yield of *β*-glucosidase from 2,146.05 U/mg protein to 8,992.67 U/mg protein.

## 4. Conclusion

The fungus *A. niger* SOI017 was found to be a good producer of *β*-glucosidase enzyme. The yield of the enzyme was enhanced substantially by optimization of the culture conditions and the medium composition, indicating that this enzyme was most probably an inducible enzyme. Concentrations of the nitrogen source (yeast extract) and carbon source (cellobiose) were found to have the most impact on the production of the enzyme. The high activity of the enzyme in an acidic pH (3–5) could find a profound use in saccharification of lignocellulosic wastes after an acidic pretreatment.

## Figures and Tables

**Table 1:** Process variables used in the central composite design (*K* = 3) with actual factor levels corresponding to coded factor levels.

Factor	code^a^	Actual factor level at coded factor levels of				
		−2	−1	0	1	2
%cellobiose	*X*_1_	0.500	1.125	1.750	2.375	3.000
%yeast extract	*X*_2_	0.050	0.275	0.500	0.725	0.950
% (NH_4_)_2_SO_4_	*X*_3_	0.6	1.1	1.6	2.1	2.6
pH	*X*_4_	3.0	4.5	6.0	7.5	9.0

^a^Code level limits are based on preliminary investigations and also to reflect what was done in practice.

*X*_1_ = (%cellobiose-1.75)/0.625, *X*_2_ = (%yeast extract-0.5)/0.225, *X*_3_ = (% (NH_4_)_2_SO_4_ -1.6)/0.5, and *X*_4_ = (pH-6.0)/1.5.

^b^Levels based on the central composite design.

**Table 2:** Treatment combinations and mean response for *β*-glucosidase production by *A. niger *SOI017.

*T*	Cellobiose (%)	Yeast extract (%)	(NH_4_)_2_SO_4_ (%)	pH	Experimental Sp activity (*μ*mol/ml/mg)
1	1.125	0.275	1.1	4.5	54.5 ± 6.3
2	1.125	0.275	1.1	7.5	6.6 ± 0.4
3	1.125	0.275	2.1	4.5	3927.0 ± 661.9
4	1.125	0.275	2.1	7.5	21.4 ± 5.3
5	1.125	0.725	1.1	4.5	3186.9 ± 68.3
6	1.125	0.725	1.1	7.5	1988.7 ± 707.2
7	1.125	0.725	2.1	4.5	2024.1 ± 263.2
8	1.125	0.725	2.1	7.5	908.4 ± 121.3
9	2.375	0.275	1.1	4.5	3.2 ± 0.1
10	2.375	0.275	1.1	7.5	4.5 ± 0.2
11	2.375	0.275	2.1	4.5	2.4 ± 0.1
12	2.375	0.275	2.1	7.5	4.5 ± 0.2
13	2.375	0.725	1.1	4.5	402.1 ± 32.4
14	2.375	0.725	1.1	7.5	146.2 ± 107.2
15	2.375	0.725	2.1	4.5	1378.6 ± 315.2
16	2.375	0.725	2.1	7.5	319.3 ± 31.6
17	3.000	0.500	1.6	6.0	445.2 ± 15.0
18	0.500	0.500	1.6	6.0	921.7 ± 93.3
19	1.750	0.950	1.6	6.0	1498.4 ± 122.7
20	1.750	0.050	1.6	6.0	3.2 ± 0.3
21	1.750	0.500	2.6	6.0	1467.9 ± 231.0
22	1.750	0.500	0.6	6.0	2677.1 ± 111.1
23	1.750	0.500	1.6	9.0	442.2 ± 18.3
24	1.750	0.500	1.6	3.0	4752.8 ± 139.2
25	1.750	0.500	1.6	6.0	1964.2 ± 265.1
26	1.750	0.500	1.6	6.0	2387.5 ± 252.1
27	1.750	0.500	1.6	6.0	2056.5 ± 33.4
28	1.750	0.500	1.6	6.0	3553.6 ± 288.4
29	1.750	0.500	1.6	6.0	1813.1 ± 114.5
30	1.750	0.500	1.6	6.0	1802.6 ± 160.3

**Table 3:** *Aspergillus* SOI017 compared with three high-score sequences of BLAST search based on ITS region.

Accession	Description	Max score	Total score	Query coverage	*E* value	Max identity
DQ778907.1	*Aspergillus niger* IBL 03110	1031	1031	100%	0.0	100%
NW_001594105.1	*Aspergillus niger* CBS 513.88	1031	3094	100%	0.0	100%
NW_001594104.1	*Aspergillus niger* CBS 513.88	1031	1031	100%	0.0	100%

**Table 4:** Analysis of variance for the evaluation of the second-order model for the model.

*Y*	Model	df	Sum of squares	Mean square	*F*	Sig.
Sp activity	Regression	14	36304923.79	2593208.84	2.608	0.038
	Residual	15	14917341.53	994489.44		

	Total	29	51222265.31		(*R* ^2^ = 0.709, *P* < .05)	

**Table 5:** Estimated regression coefficients for *β*-glucosidase production.

Model	Parameter estimates	*P* value
Constant	−5927.29	.430
%cellobiose	2266.84	.433
%yeast extract	17046.43	.039
%amm sulfate	5762.06	.134
pH	−375.86	.776
%cellobiose × %yeast extract	−829.59	.646
%cellobiose × %amm sulfate	−98.28	.903
%cellobiose × pH	331.26	.233
%yeast extract × %amm sulfate	−2829.54	.231
%yeast extract × pH	59.00	.937
%amm sulfate × pH	−381.47	.269
%cellobiose × %cellobiose	−1258.96	.020
%yeast extract × %yeast extract	−9695.37	.020
%amm sulfate × %amm sulfate	−579.77	.457
pH × pH	−6.08	.944

**Table 6:** The model equations for kinetic parameters.

*Y*	Model equations	*R* ^2^	*P* value
Sp activity	*Y* = 2266.841*X*_1_ + 17046.43*X*_2_ + 5762.059*X*_3_- 375.861*X*_4_- 829.59*X*_1_ *X*_2_- 98.276*X*_1_ *X*_3_ + 331.259*X*_1_ *X*_4_- 2829.54*X*_2_ *X*_3_+ 58.998*X*_2_ *X*_4_- 381.471*X*_3_ *X*_4_- 1258.96*X*_1_^2^- 9695.37*X*_2_^2^ – 579.765*X*_3_^2^ – 6.083*X*_4_^2^ -5927.29	0.709	.038

**Table 7:** Model predicted value for specific activities at optimum condition.

CON	%cellobiose	%yeast extract	% (NH_4_)_2_SO_4_	pH	Sp activity (umol/ml/mg protein)	
					Predicted	Experimental
1	1.750	0.500	1.6	3	3568.1	3793.3
2	1.750	0.275	2.1	3	3489.9	7957.1
3	1.750	0.275	2.6	3	3961.3	8046.9
4	1.125	0.275	2.1	3	3985.8	7898.1
5	1.125	0.275	2.6	3	4487.9	8992.7
6	1.125	0.600	2.6	3	4634.1	4615.4
